# Cancer‐related pain: A bidirectional modulator of cancer progression

**DOI:** 10.1002/ctm2.70817

**Published:** 2026-09-11

**Authors:** Junzhe He, Yu Zhang, Qian Liu, Biao Yan, Peiwei Chai, Renbing Jia

**Affiliations:** ^1^ Department of Ophthalmology Ninth People's Hospital, Shanghai Jiao Tong University School of Medicine Shanghai People's Republic of China; ^2^ Shanghai Key Laboratory of Orbital Diseases and Ocular Oncology Shanghai People's Republic of China; ^3^ Department of Ophthalmology Shanghai General Hospital, Shanghai Jiao Tong University School of Medicine Shanghai People's Republic of China

**Keywords:** bidirectional crosstalk, cancer‐related pain, cancer neuroscience, neoplasm, neuroimmunology, therapeutic implications, tumour microenvironment

## Abstract

**Background:**

Cancer‐related pain (CRP), with a high prevalence and prognostic value in malignancies, is not only a passive phenomenon but rather engages in a bidirectional crosstalk with cancer progression.

**Main Topics:**

This review focuses on the intricate reciprocal relationship between CRP and cancer progression, delineating how advanced cancers promote CRP directly through tissue destruction, invasion and inflammation, or indirectly via inflammatory, neural, stromal and metabolic components within the tumour microenvironment. However, controversies remain in the field, such as the ambiguous role of sensory neurons in cancer progression and the potential pro‐tumorigenic risk of opioids. Notably, CRP regulates cancer progression through local pain‐associated mediators (e.g., CGRP, SP), the pain‐stress axis, central nuclei‐mediated peripheral immune modulation, sensory‐sympathetic/vagal circuits and direct effects on tumour biology.

**Conclusion:**

Future research should further elucidate the mechanistic interplay between pain and cancer, as targeting CRP may offer novel therapeutic opportunities for anti‐cancer treatment.

**Highlights:**

- CRP and tumour progression engage in a dynamic, bidirectional circuit mediated by multiple mechanisms in neuroimmunology and cancer neuroscience.
- Analgesic interventions may affect tumour progression, while anticancer therapies can influence pain.
- Targeting pain in cancer may have promising therapeutic value in cancer progression.

## Graphical abstract

CRP and tumour progression engage in a dynamic, bidirectional circuit mediated by multiple mechanisms involving neuroimmunology and cancer ‐neuroscience. Analgesic interventions may affect tumour progression, while anticancer therapies can influence pain. Targeting pain in cancer may have promising therapeutic value in cancer progression.

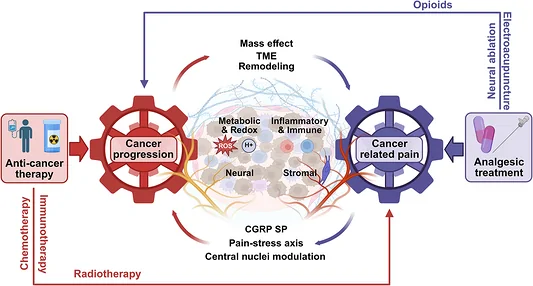

## 1 BACKGROUND

Cancer‐related pain (CRP) is a prevalent and debilitating symptom among patients with malignancy, with a pooled prevalence of 44.5% reported between 2014 and 2021.^1^ Beyond its clinical burden, CRP is increasingly recognized as a significant prognostic factor for survival.^2^, ^3^ Notably, effective pain management has been associated not only with symptomatic relief but also with prolonged overall survival (OS),^4^ suggesting an intricate biological interplay between nociceptive signalling and tumour progression.

Historically, CRP was regarded as a passive consequence of advanced disease. However, emerging evidence reveals a more intricate relationship: cancer progression and pain are not unidirectionally linked but engage in dynamic, bidirectional crosstalk mediated by molecular signalling, neuro‐immune and neuro‐cancer interactions and microenvironmental remodelling.^5^, ^6^, ^7^, ^8^

Current research into the interplay between cancer progression and pain has yielded valuable insights, yet these findings are often presented in fragmented ways across individual molecular pathways or context‐specific models, highlighting the need for a unified conceptual framework that captures the full complexity of their bidirectional relationship. While several studies suggest a tumour‐promoting role for nociceptive signalling, other studies have indicated context ‐ dependent anti‐tumour effects of sensory neurons.^9^, ^10^, ^11^ Compounding this complexity, growing concerns emerge about the oncological safety of conventional analgesics (particularly opioids).^12^, ^13^, ^14^ Given the rapid expansion of mechanistic insights and the emerging clinical dilemmas surrounding pain management in oncology, a timely and integrative review is urgently needed.

Here, we provide a comprehensive review that: (1) delineates how cancer progression drives pain; (2) summarizes evidence that pain signalling actively influences cancer progression; (3) discusses the bidirectional interplay between anticancer and analgesic therapies; and (4) explores novel therapeutic methods for CRP and cancer progression.

By integrating fragmented findings, this review aims to provide a cohesive framework for understanding the neurobiological interface between cancer and pain, as well as for developing strategies that alleviate pain and improve oncological outcomes in the future.

## 2 CANCER‐RELATED PAIN: PERIPHERAL NOCICEPTION AND CENTRAL SENSITIZATION

Pain signalling generally begins when peripheral nociceptors detect noxious mechanical, thermal, or chemical stimuli and convert them into neural signals that are transmitted through primary afferents and dorsal root ganglia (DRG) to the spinal dorsal horn and higher brain centers.^15^, ^16^ In cancer, this pathway can be persistently activated by tumour growth, tumour‐stromal‐immune cell interactions, local acidosis, bone remodelling, and treatment‐related neuropathy, making cancer pain a mixed state involving inflammatory, neuropathic and cancer‐specific mechanisms.^17^, ^18^ At the peripheral level, tumour‐derived and TME‐derived mediators can activate or sensitize receptors and ion channels on sensory neurons, lowering nociceptor thresholds and promoting peripheral sensitization and hyperalgesia.^17^, ^18^, ^19^ Nerve injury further contributes to cancer pain by inducing changes in sodium, calcium channel expression, ectopic spontaneous activity, Aβ‐fibre plasticity, and interactions among Schwann cells (SCs), glial cells, neurons and immune cells, increasing primary afferent excitability and peripheral sensitization.^20^, ^21^ Sustained peripheral input may then drive central sensitization.^6^, ^22^, ^23^ In cancer pain models, these central changes are also associated with spinal neurochemical reorganization and glial activation, which may help maintain hyperalgesia and persistent pain.^24^

### 2.1 Tumour progression: Mechanical invasion and tissue destruction

One of the most direct routes by which cancer progression causes pain is through tumour growth itself. Expanding tumours may compress, infiltrate, or distort adjacent structures, including nerves, bones, viscera, pleura, chest wall, and soft tissues.^18^ For instance, patients with lung cancer may suffer from chest pain due to tumour‐induced compression of the chest wall or intercostal nerves.^25^, ^26^, ^27^ Retroperitoneal tumours affecting the lumbar or abdominal nerve plexus could result in lower back or abdominal pain.^28^, ^29^ Brain tumours might induce headaches and neuralgia through compression of surrounding brain tissue.^30^

Tumour progression may also impair organ function and indirectly cause pain. Cervical cancer, for instance, may obstruct the urinary tract, leading to hydronephrosis and severe back pain.^31^

In addition, tumour growth may lead to tissue necrosis and infection, triggering inflammatory responses that further aggravate pain. Conditions such as cancer‐induced bowel obstruction or liver tumour‐associated necrosis can result in acute abdominal pain and systemic inflammation.^32^, ^33^

Bone metastasis is another important cause of cancer pain. In cancer‐induced bone pain (CIBP), tumour growth disrupts bone homeostasis and promotes osteoclast‐mediated bone destruction. This process can generate structural instability, acidic microenvironments, inflammatory signalling and sensitization of peripheral sensory neurons, which together contribute to CIBP.^34^, ^35^

### 2.2 The nociceptive TME: A multidimensional hub of inflammatory, neural, stromal and metabolic stressors

Tumour progression is accompanied by alterations within the TME.^36^ Beyond its role in tumour progression, the TME may also directly or indirectly modulate nociceptive pathways. Key components include inflammatory mediators, immune cells, neural elements, neurotrophic factors, stromal remodelling and metabolic changes.

#### 2.2.1 Inflammation and immune‐related mechanisms

A systematic review of 25 studies in bone cancer pain models identified inflammatory cytokines such as TNF‐α, IL‐1β, IL‐6, IL‐17, IL‐18 and IL‐33 as contributors to pain behaviours.^37^ For example, in a rat model of bone cancer pain, blockade of TNFR1 or IL‐6R attenuated mechanical and thermal hyperalgesia, supporting a functional role of TNF‐α and IL‐6 signalling in CIBP.^38^ In pancreatic ductal adenocarcinoma (PDAC), neuroinflammation and tumour‐nerve interactions, particularly perineural invasion (PNI), are closely associated with pain.^39^ In HNSCC and oral cancer, inflammatory mediators and nociceptor sensitization may contribute to cancer pain,^40^, ^41^ and TNF‐α inhibition reduced nociception in oral cancer models.^42^

Clinically, elevated TNF‐α, IL‐1 and IL‐6 have been correlated with cancer pain severity in non‐small cell lung cancer (NSCLC) patients.^43^ In patients with early‑stage breast cancer, pain was significantly associated with elevated levels of IL‑13, IL‑7 and C‑reactive protein,^44^ while a systematic review also supported a link between IL‐6 and cancer pain across tumour types.^45^

Tumour progression, tissue damage, or immune cells within the TME may lead to the generation of inflammatory mediators.^6^, ^46^ Mechanically, these inflammatory mediators may further interact with corresponding receptors on peripheral nociceptors, triggering a series of events that upregulate relevant ion channels,^19^ ultimately leading to hyperalgesia.^18^ Taken together, these findings support inflammatory signalling as a critical element of CRP, yet it remains to be further clarified whether inflammation serves as a common pathogenic mechanism and a unifying target for analgesic therapy under pan‐cancer settings.

Immune cell involvement is increasingly recognized but less definitively established in cancer pain. Macrophage‐to‐neuron‐like transition (MNT) has been reported in lung carcinoma using single‐cell RNA sequencing and has been linked to nocifensive behaviour in animal models.^47^ Another recent study revealed that macrophage depletion reduces CIBP in lung cancer bone metastasis models,^48^ while Th17/Treg imbalance exacerbates pain via microglial activation in a bone cancer model.^49^ In an osteosarcoma model, β2‑ and β3‑adrenergic receptors play a role in the development of cancer pain possibly by modulating neural macrophages.^50^ A single‑cell sequencing study on lung adenocarcinoma further revealed that tumour‑infiltrating DCs upregulate pain‑related genes, with their corresponding receptors present on DRG neurons, hinting at a possible DC‑mediated pro‑nociceptive effect through secreted factors.^51^ Nevertheless, most evidence seems to be correlative, whereas definitive evidence supporting a causal contribution of immune cells alone remains lacking.

#### 2.2.2 Neural components, neurotrophic factors and tumour‐associated neurogenesis

Neural remodelling is another important TME‐associated mechanism in CRP, especially in tumours with significant nerve involvement. In multiple CIBP models of breast cancer or prostate cancer,^52^, ^53^, ^54^ the sensory fibres were significantly increased in the peri‐tumour region. Notably, in these models, hyperalgesia was observed.

In pancreatic cancer, PNI and sensory fibres were considered to be associated with pain severity, reduced OS and relapse free survival (RFS).^55^, ^56^ In patients with HNSCC, PNI was associated with pain and altered extracellular matrix programs^41^ and experimental models of oral cancer have indicated a correlation between spontaneous nociception and PNI.^40^ Mechanistically, PNI‐associated pain may be attributed to peripheral nerve injury and nociceptor sensitization.^40^ These findings suggest that PNI represents a context‐dependent prominent mechanism of cancer pain.

SCs are emerging regulators of tumour‐nerve interactions, although direct evidence linking SCs to cancer pain generation remains limited. They may produce neuroactive and inflammatory mediators, responding to tumour‐derived signals.^57^ SCs can be activated by tumour‐derived inflammatory signals, and this activation has been associated with nociceptive signalling in oral squamous cell carcinoma (OSCC) models.^42^ In PDAC, SCs facilitate PNI and tumour‐nerve remodelling, which is considered to correlate with pain severity.^58^, ^59^

Collectively, current evidence implies a model in which SCs primarily contribute to cancer progression and tumour‐nerve interactions, with their involvement in cancer pain being indirect and context‐dependent, possibly through inflammatory signalling and PNI rather than direct nociceptive induction.

Nerve growth factor (NGF) plays a key role in cancer pain, particularly in bone metastasis and PNI‐associated tumours. In breast cancer bone metastasis models, NGF promotes sensory nerve sprouting, while NGF blockade reduces pain behaviours.^53^ Clinically, anti‐NGF antibody, tanezumab, improves pain outcomes in bone metastasis patients at early time points, although long‐term efficacy is limited and adverse events occur.^60^ In patients with pancreatic cancer, NGF/ Tropomyosin receptor kinase A (TrkA) signalling correlates with PNI, pain severity, and prognosis.^61^, ^62^, ^63^, ^64^ In OSCC models, anti‐NGF reduced pain‐related behaviour and tumour proliferation.^65^

#### 2.2.3 Angiogenesis and stromal remodelling

The correlation between angiogenesis and CRP has been increasingly recognized. Vascular endothelial growth factor (VEGF) A/VEGFR blockade reduces hyperalgesia in breast and lung cancer bone pain models,^66^, ^67^ while exogenous VEGFA at doses comparable to TME concentration^68^ may also induce hyperalgesia.^69^ However, vascular mechanisms remain context‐dependent and are most strongly supported in bone metastatic pain, requiring further validation before generalization to broader cancer pain mechanisms.

Cancer‐associated fibroblasts (CAFs) have been implicated in the promotion of cancer pain, with the evidence to date coming from pancreatic cancer. In PDAC, nociceptor neurons interact with CAFs through calcitonin gene‐related peptide (CGRP)‐NGF signalling. This interaction suppresses CAF‐derived IL‐15, reduces NK‐cell infiltration, and promotes both PDAC progression and cancer pain.^56^ CGRP has been identified as a mediator of oral cancer pain, and CGRP receptor components are detected in fibroblast‐ and immune‐marker‐expressing cells in the TME.^70^ However, these findings indicate possible CAFs’ involvement in nociceptive signalling, whether CAF is a sufficient driver of cancer pain remains to be further explored.

#### 2.2.4 Metabolic and redox stressors

Tumour tissues frequently exhibit an acidic microenvironment, with pH values ranging from 6.0 to 6.9,^71^, ^72^ which may induce the activation of acid‐sensing ion channels (ASICs). Direct evidence for this mechanism comes most prominently from CIBP. In a tibia CIBP model of myeloma,^73^ inhibition of ASIC3 has been shown to exert pain‐relieving effects.

Extracellular P2X (belongs to purinergic receptor) signalling represents another potential mechanism underlying cancer pain. Extracellular ATP released from tumour cells within the TME serves as an important mediator of cancer pain, which may activate P2X receptors on nociceptive neurons and glial cells, leading to neuronal hyperexcitability, peripheral sensitization, enhanced nociceptive transmission and central sensitization.^74^ Inhibition of P2 × 3 in the DRG demonstrated preliminary effects in mitigating bone cancer pain in a mouse model.^75^ Notably, P2 × 7 is expressed in various cancer types, including colorectal cancer (CRC),^76^ prostate cancer,^77^ breast cancer^78^ and neuroblastoma,^79^ and inhibition of P2 × 7 also showed preliminary effects in reducing bone cancer pain.^80^

Oxidative stress may also be elevated in the TME and could be enhanced by chemotherapy.^81^, ^82^ In fact, key terminal products or byproducts of oxidative stress have been identified as endogenous regulators of transient receptor potential ankyrin 1 (TRPA1) channel expressed on nociceptive receptors.^83^ Antioxidants and TRPA1 antagonists reduce hyperalgesia in melanoma and breast cancer bone metastasis models, as well as in chemotherapy‐induced pain models.^84^, ^85^, ^86^, ^87^

#### 2.2.5 Extracellular vesicles: Emerging mechanism

Cancer‐derived extracellular vesicles (EVs) represent another emerging mechanism of cancer–neuron communication. In HPV‐positive HNSCC models, tumour‐derived small EVs were shown to communicate with TRPV1‐positive neurons, leading to hyperalgesia in mice.^88^ Similar findings have been reported in oral cancer models, where vesicles carry pain mediators.^89^

### 2.3 Central sensitization and the chronification of cancer pain

Persistent peripheral pain produces sustained nociceptive input to the spinal cord, resulting in central sensitization that may contribute to the amplification of pain signalling within the spinal cord and brain beyond tumour sites.^17^, ^90^ Mechanically, glutamate and glutamatergic N‐methyl‐D‐aspartate (NMDA) signalling, innate immune responses and glial cells within the central nervous system (CNS) may play roles in this process.

Studies of bone cancer pain provide direct experimental examples of these mechanisms. In male mice bearing tibial tumours, inhibition of spinal absent in melanoma 2 (AIM2, a protein) increased STING expression, reduced expression of the NMDA‐receptor subunit GluN1, and attenuated mechanical allodynia, linking innate immune signalling to excitatory glutamatergic transmission.^91^ In an osteosarcoma model, secondary mechanical hyperalgesia was accompanied by increased microglial staining in the spinal dorsal horn at an earlier stage and increased astrocytic staining at a later stage, while no significant alterations in the capsaicin‐sensitive nociceptive fibres were found.^24^ Intrathecal knockdown of microglial P2 × 4R attenuated CIBP‐related nociceptive behaviours and reduced TLR4/p38 MAPK activation as well as BDNF and TNF‐α release in a rat model, suggesting that microglial purinergic signalling may contribute to spinal sensitization in bone cancer pain.^92^ Similarly, selective knockdown of astrocyte‐derived IL‐17A attenuated the development of mechanical allodynia and thermal hyperalgesia in mice with bone cancer pain, whereas astrocytic IL‐17A overexpression induced pain hypersensitivity possibly through CaMKIIα signalling.^93^

Although the most direct mechanistic evidence for central sensitization in cancer pain comes from bone cancer pain models, this concept may extend beyond bone cancer and bone metastasis. Reviews of cancer pain and cancer survivorship increasingly describe central sensitization as a clinically relevant mechanism in pancreatic cancer,^94^ HNSCC^95^ and CIPN.^96^ However, compared with bone cancer pain, whether central sensitization is a widespread phenomenon and shares common mechanisms across all cancer types remains to be further validated by both clinical and experimental studies.

**FIGURE 1:**
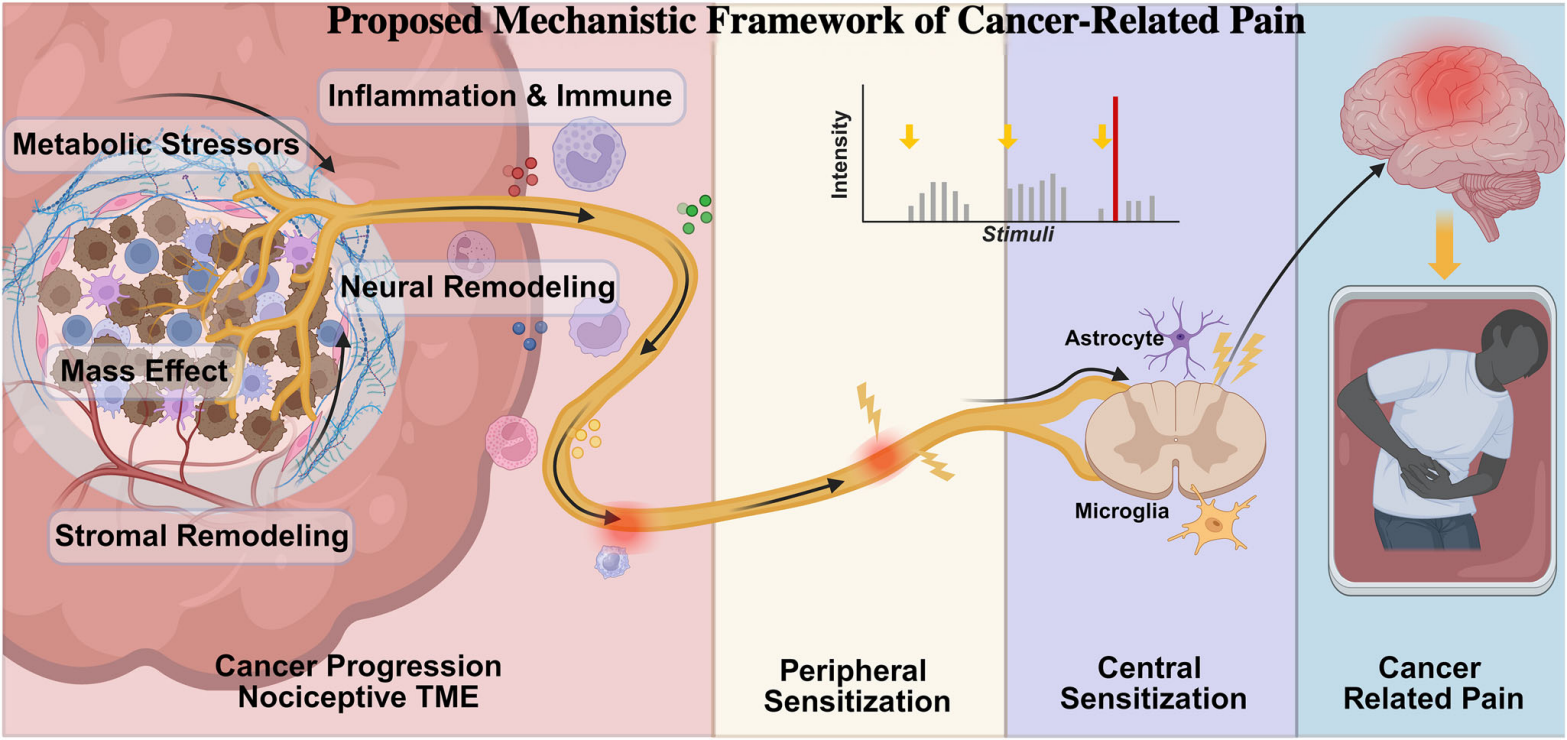
Proposed mechanistic framework of cancer‐related pain. Peripheral nociceptors may be activated or sensitized by tumour or TME related factors, leading to peripheral sensitization and sustained nociceptive input may drive central sensitization. CGRP, calcitonin gene‐related peptide; SP, substance P; TME, tumour microenvironment. Original figure created with BioRender.com.

Figure 1 illustrates a proposed framework for cancer pain mechanisms, drawn from evidence that appears to apply to selected cancer types. Cancer pain, however, is a multifactorial and complex process whose underlying mechanisms remain incompletely explored. Thus, this figure should be viewed as a working model rather than a definitive map. Further studies are needed to determine whether these mechanisms are shared across multiple cancers, whether they map onto specific features of the TME, and whether they are inter‐linked through clear upstream and downstream relationships. Mechanisms of CRP in different cancer types are summarized in Table 1.

**TABLE 1:** Mechanisms of CRP in different cancer types.

Cancer type	Implicated mediators	Key findings (copied from original article)	Evidence strength	Ref
Bone cancer	TNF‐α, IL‐6	Blockade of TNFR1 or IL‐6R attenuated mechanical and thermal hyperalgesia TNF‐α and IL‐6 regulate bone cancer pain via TRPA1 signal pathway	Animal experiment	^38^
PDAC	PNI / Neurogenic inflammation	PNI (cancer cell invading nerves) → neurogenic inflammation → eventually contribute to cancer pain	Reviews	^39^, ^59^
Oral cancer	TNF‐α	Targeting TNF‐α significantly attenuated nociception	Animal experiment	^42^
NSCLC	TNF‑α, IL‑1, IL‑6	The levels of tumour necrosis factor‑α (TNF‑α), interleukin‑1 (IL‑1), and interleukin‑6 (IL‑6) were found to correlate with the degree of cancer pain	Clinical correlation	^43^
Early‑stage breast cancer	IL‑13, IL‑7, CRP	Pain was significantly associated with elevated levels of IL‑13, IL‑7 and CRP	Clinical correlation	^44^
Lung carcinoma	Neuron‐like TAM	Macrophage to neuron‐like cell transition is associated to nocifensive behaviour	Correlation in animal model	^47^
Lung cancer bone metastasis	Macrophage	Deletion of macrophage significantly attenuated CIBP	Animal experiment	^48^
bone cancer	Ratio of Th17 and Treg	Imbalanced ratio of Th17 and Treg aggravated pain	Correlation in animal model	^49^
Lung adenocarcinoma	DCs	Tumour‑infiltrating DCs may upregulate pain‑related genes, with their corresponding receptors present on DRG neurons	Observations in clinical samples	^51^
Pancreatic cancer	Sensory fibres	Ablation of nociceptor neurons alleviates cancer pain	Correlation in animal model	^56^
Breast cancer bone metastasis	Sensory fibres	Sensory fibres were significantly proliferated in the peri‐tumour region with hyperalgesia observed	Correlation in animal model	^53^
Prostate cancer bone metastasis	Sensory fibres	Sensory fibres were significantly proliferated in the peri‐tumour region with hyperalgesia observed	Correlation in animal model	^54^
HNSCC	PNI	PNI was associated with pain	Clinical correlation	^41^
Oral cancer	PNI	Mice with PNI exhibited spontaneous nociception and mechanical allodynia	Animal experiment	^40^
Breast cancer bone metastasis	NGF	Blocking NGF inhibited pathological sensory nerve sprouting and attenuated cancer pain‐related behaviours	Animal experiment	^53^
Bone metastasis	NGF	The anti‐NGF antibody tanezumab produced a statistically greater improvement in pain at Week 8 than placebo	Clinical trial	^60^
PDAC	NGF	Higher levels of NGF and TrkA (NGF receptor) correlated with more severe pain, PNI, and poorer prognosis	Clinical correlation	^62^, ^63^, ^64^
Oral cancer	NGF	Anti‐NGF reduced pain‐related behaviour	Animal experiment	^65^
Oral cancer	BDNF	Neutralization of BDNF or inhibition of TrkB reversed tumour‐induced pain‐like behaviours	Animal experiment	^97^
Bone cancer	GDNF	Normalizing the expression of GDNF in the spinal cord attenuated cutaneous hyperalgesia	Animal experiment	^98^
Breast cancer bone metastasis	VEGFA/VEGFR	Blockage of VEGFA or VEGFR significantly reduced tumour‐induced hyperalgesia	Animal experiment	^66^
Lung cancer bone metastasis	VEGFA/VEGFR	Blockage of VEGFA or VEGFR significantly reduced tumour‐induced hyperalgesia	animal experiment	^67^
/	VEGFA	Intraplantar injection of VEGFA into the hind paws of mice at doses comparable to those present in the tumour microenvironment (based on breast, prostate, colorectal and other cancers) induced significant hyperalgesia responses	Animal experiment	^69^
OSCC	SCs	SCs can be activated by tumour‐derived TNF‐α, and this activation has been associated with nociceptive signalling	Animal experiment	^42^
PDAC	CAFs	Nociceptor neurons interact with cancer‐associated fibroblasts, which suppresses CAF‐derived IL‐15, reduces NK‐cell infiltration and cytotoxicity, and thus promotes both PDAC progression and cancer pain	Animal experiment	^56^
Myeloma CIBP	ASIC3, acidic environment	Inhibition of ASIC3 has been shown to exert pain relieving effects	Animal experiment	^73^
Melanoma bone metastasis	Oxidative stress	The application of antioxidants or TRPA1 antagonists effectively alleviated hyperalgesia	Animal experiment	^84^
Breast cancer bone metastasis	Oxidative stress	The application of antioxidants or TRPA1 antagonists effectively alleviated hyperalgesia	Animal experiment	^85^
HNSCC	sEVs	sEVs were shown to communicate with TRPV1‐positive neurons Injection of purified sEVs lead to hyperalgesia in mice	Animal experiment	^88^
Oral cancer	sEVs	Extracellular vesicles released by cancer cells can carry pain mediators and induce hyperalgesia	Animal experiment	^89^

Abbreviations: ASIC3, acid‐sensing ion channel 3; BDNF, brain‐derived neurotrophic factor; CAFs, cancer‐associated fibroblasts; CIBP, cancer‐induced bone pain; CRP, C‐reactive protein; DCs, dendritic cells; DRG, dorsal root ganglia; GDNF, glial cell line‐derived neurotrophic factor; HNSCC, head and neck squamous cell carcinoma; NGF, nerve growth factor; NSCLC, non‐small cell lung cancer; PDAC, pancreatic ductal adenocarcinoma; PNI, perineural invasion; SCs, Schwann cells; sEVs, small extracellular vesicles; TAM, tumour‐associated macrophages; TRPA1, transient receptor potential ankyrin 1; TRPV1, transient receptor potential vanilloid 1; VEGF, vascular endothelial growth factor.

## 3 PAIN AS A MODULATOR OF MALIGNANCY: DECIPHERING THE AFFERENT NEURO‐TUMOUR SIGNALLING AXIS

### 3.1 Structural basis: Aberrant intertumoral sensory neurogenesis and hyperinnervation

Innervation is increasingly recognized as an important form of crosstalk between cancer and the nervous system.^99^ Cancer cells can activate neural pathways, promoting the recruitment and remodelling of peripheral nerves within the TME.^100^ Sensory nerve proliferation has been observed in the peri‐tumoral regions and is closely associated with CRP. Models of CIBP in breast and prostate cancer show marked sensory fibre proliferation accompanied by hyperalgesia.^52^, ^53^, ^54^ These findings suggest that sensory innervation may provide a common basis for both CRP and cancer progression.^8^ Clinically, increased sensory innervation in pancreatic cancer correlates with more severe pain, shorter OS and reduced RFS.^55^, ^56^

Despite these observations, the relationship between sensory neurons, pain and tumour progression remains controversial. Opposing effects of sensory neuron modulation have been reported even in the same cancer type (Table 2).

**TABLE 2:** Controversial findings regarding the role of sensory nerves in cancer.

Cancer type	Interventions	Effects	Role	Ref
Melanoma	Genetic ablation of TRPV1+ Sensory neurons	1.Increase number and proportion of tumour infiltrating CD8+ T cells 2.Reduced proportion of PD‐1+LAG3+TIM3+ CD8+ T cells	Pro‐tumour	^101^
Melanoma	1. First RTX treatment 2. Second cancer inoculation	1. Reduce tumour growth 2. Enhance CD8+T cell function, reduce CD8+T cell exhaustion	Pro‐tumour	^101^
Melanoma	RAMP1 KO in CD8+T	Attenuated CGRP induced CD8+T cell Exhaustion and cytotoxicity reduction	Pro‐tumour	^101^
Melanoma	1. First cancer inoculation 2. Second RTX treatment	Inhibit angiogenesis and cancer Progression	Pro‐tumour	^9^
Melanoma	1. First RTX treatment 2. Second cancer inoculation	Promote angiogenesis and cancer progression	Anti‐cancer	^9^
Melanoma	Genetic depletion of Nav1.8+ sensory fibres	Promote angiogenesis and cancer progression	Anti‐cancer	^9^
Melanoma	Over activate sensory neurons using DREADD	Inhibit angiogenesis and cancer progression	Anti‐cancer	^10^
Melanoma	Inhibit sensory neurons' activity using DREADD	Promote angiogenesis and cancer progression	Anti‐cancer	^10^
Melanoma	In silicon analysis	A positive correlation between sensory neuron related genes and prognosis	Anti‐cancer	^10^
Breast cancer	Co‐injection of sensory neuron from mouse DRG and human TNBC cells	Facilitate metastasis	Pro‐tumour	^102^
Breast cancer	Co‐culture of sensory neurons and TNBC cells	1. Cancer cells could attach to nerve fibres with a higher migration velocity 2. Upregulated expression of genes concerning migration and adhesion in cancer cells	Pro‐tumour	^102^
Breast cancer^a^	TRPV1 antagonists and genetic deletion of TRPV1	1. Decreased HGF production in sensory neurons 2. Inhibit bone colonization, bone pain and distant metastasis	Pro‐tumour	^103^
Breast cancer	Activating TRPV1 on sensory neurons using olvanil	1. Elevating the CD8+T infiltration 2. Inhibit breast cancer metastasis	Anti‐cancer	^104^

Abbreviation: DREADD, Designer Receptors Exclusively Activated by a Designer Drug.

^a^Breast cancer cells in this experiment were inoculated into the bone

In 2020, genetic depletion of Nav1.8^+^ sensory neurons was reported to promote angiogenesis and progression in a melanoma mouse model.^9^ To avoid compensatory effects of genetic ablation and the cytotoxicity of resiniferatoxin (RTX), the same group of researchers subsequently adopted Designer Receptors Exclusively Activated by Designer Drugs (DREADDs) to reversibly modulate sensory neuronal activity while preserving neuronal integrity, and activation of sensory neurons was associated with angiogenesis and tumour growth.^10^ These findings suggested an inhibitory role of sensory neurons in melanoma.

In contrast, a 2022 Nature study reported an opposite finding. Using genetic ablation of TRPV1^+^ sensory neurons, RTX treatment (prior to tumour inoculation), and CGRP antagonists, this study demonstrated that nociceptors promote melanoma progression by suppressing anti‐tumour immunity through the CGRP receptor activity modifying protein 1 (RAMP1) axis, leading to immune exhaustion.^101^

Through a detailed analysis and comparison of these studies, we propose that several factors may account for these discrepancies. First, different studies may emphasize distinct functions of sensory neurons or different nociceptive neuropeptides. Tumour‐promoting studies have focused on the immunosuppressive effects of CGRP, whereas tumour‐suppressive studies have highlighted the anti‐angiogenic effects of sensory neurons, suggesting that these findings reflect complementary rather than mutually exclusive mechanisms.

Second, methodological differences may also contribute. Although DREADDs enable precise modulation of neuronal activity, they may generate centrally active metabolites,^105^ raising the possibility of CNS involvement. Given the recognized role of the CNS in cancer progression,^106^ such effects might need to be considered when interpreting chemogenetic studies.

Conflicting findings have also been reported in breast cancer.^102^, ^103^, ^104^ Loss of vagal afferent fibres may partly explain these differences, as vagotomy promotes metastasis in breast cancer models.^107^

### 3.2 Peripheral effector repertoire: From classical neuropeptides (CGRP/SP) to emerging non‐canonical mediators

TRPV1 positive neurons may play significant roles in pain signalling. In adults, they mainly consist of unmyelinated peptidergic neurons.^108^ Upon activation, these neurons release glutamate and neuropeptides,^109^ among which CGRP and SP have been extensively studied.^110^ In fact, in pancreatic cancer, sensory neurons sensitized by cancer‐cell‐derived NGF could subsequently secrete neurotransmitters, CGRP and SP back into the tumour.^111^ A concise overview of key findings regarding their roles in cancer progression is provided in Table 3.

**TABLE 3:** CGRP, SP and cancer progression.

Mediators	Cancer types	Major impact	Mechanism	Ref
CGRP	OSCC	Pro‐tumour	Inhibit CD4+ T, CD8+T and NK cells	^112^
	MTC	Pro‐tumour	CGRP‐RAMP1 (in DCs)→elevated KLF2→hamper DC development→impair anti‐tumour immunity	^113^
	HNSCC	Pro‐tumour	Suppress Th1 CD4+T and CD8+T	^114^
	Pancreatic cancer	Pro‐tumour	CGRP and NGF→inhibit CAFs from releasing IL‐15→inhibit NK cells	^56^
	Melanoma	Pro‐tumour	CD8+T cell exhaustion	^101^
	OSCC	Pro‐tumour	ERK and YAP	^115^
	Gastric cancer	Pro‐tumour	PI3K‐AKT and CaMK pathways →Rb→E2F→metastasis and proliferation	^116^
	Oral mucosa carcinoma	Pro‐tumour	Low glucose→cancer cell secreting NGF→nociceptive neuron derived CGRP→Rap1→undermine mTOR‐Raptor interaction→protective autophagy	^117^
	TNBC	Pro‐tumour	CGRP‐RAMP (in CAF)→facilitate myCAF phenotype→ collagen deposition and immune exclusion	
SP	HNSCC, HGSOC	Pro‐tumour	Phosphorylation of ERK1/2→cancer cell proliferation	^118^
	Colorectal and ovarian cancer	Pro‐tumour	MMP‐2/9→cancer cell migration	^119^, ^120^
	Pancreatic cancer	Pro‐tumour	Akt→NF‐kB→proliferation, migration	^121^
	Gallbladder cancer	Pro‐tumour	Akt→NF‐kB→proliferation, migration	^122^
	Cervical cancer	Pro‐tumour	ERK1/2 and MMP‐9→proliferation and invasion	^123^
	Gastric cancer	Pro‐tumour	Increase calcium concentration within cancer cell	^124^
	Ovarian cancer	Pro‐angiogenic	Upregulate VEGF and VEGFR	^119^
	Breast cancer	Pro‐tumour	Induce death of high NK1R cancer cell→releasing ssRNA→TKR7 on cancer cell→promoting metastatic oncogenes	^11^
	Melanoma	Anti‐tumour	Facilitate NK and T cell	^125^
	Breast cancer	Anti‐tumour	Death of cancer cells with high NK1R expression	^11^

Abbreviations: CGRP, calcitonin gene‐related peptide; DAMPs, damage‐associated molecular patterns; HCC, hepatocellular carcinoma; HGSOC, high‐grade serous ovarian carcinoma; HMGB1, high‐mobility group box 1; HNSCC, head and neck squamous cell carcinoma; KLF2, Krüppel‐like factor 2; MTC, medullary thyroid cancer; NSCLC, non‐small cell lung carcinoma; OSCC, oral squamous cell carcinoma; RAGE, receptor for advanced glycation end products; SP, substance P; TLR, toll‐like receptor.

In addition to these well‑defined nociceptive neuropeptides, several emerging molecules may also play a role in cancer pain. However, they are not yet well‑established within a unified ‘pain‐molecule‐tumour progression’ axis, and direct evidence remains scant.

#### 3.2.1 Classical nociceptive neuropeptides

CGRP and substance P (SP) are both well‐established pain‐related neurotransmitters.^7^ In addition, a substantial body of evidence supports that, under tumour conditions, sensory neurons can secrete CGRP and SP, thereby promoting cancer progression.^11^, ^101^

Current evidence suggests a predominantly pro‐tumour role of CGRP in several cancer types, although it exerts effects through mechanistically distinct pathways in different biological contexts.

##### CGRP

Accumulating evidence suggests that CGRP may promote tumour progression by compromising anti‐tumour immune responses. Broad RAMP1 expression within the TME^126^, ^127^ suggests that CGRP may influence multiple TME components. In melanoma models, CGRP promotes CD8+ T cell exhaustion, whereas RAMP1 deficiency protects CD8+ T cells from exhaustion.^101^ CGRP also inhibits dendritic cells (DCs) development^113^ in medullary thyroid cancer models and limits T cell and NK cell infiltration in OSCC^112^ models. In pancreatic cancer models, nociceptor‐derived CGRP and NGF regulate CAFs and suppress IL‐15 release, impairing NK‐cell infiltration and cytotoxicity.^56^ In triple negative breast cancer (TNBC) models, CGRP‐RAMP1 signalling promotes myCAF activation, collagen deposition and immune exclusion.^128^

CGRP may also directly act on cancer cells. In gastric cancer models, sensory nerve‐derived CGRP enhances tumour cell calcium signalling, facilitating metastasis and progression.^116^ Under low glucose conditions, oral mucosa carcinoma cells induce NGF release through reactive oxygen species (ROS)‐dependent pathways, which promotes neuronal CGRP production. CGRP may support protective autophagy by disrupting mTOR‐Raptor interaction through Rap1.^117^ Thus, CGRP may function not only as an immunomodulatory neuropeptide, but also as a stress‐adaptive signal that helps cancer cells survive metabolic stress.

Nevertheless, CGRP may not be viewed as uniformly pro‐tumorigenic. In non‐cancerous inflammatory settings, extracellular CGRP enhances Th1 differentiation through CREB, ATF3 and STAT1 signalling, and may promote T cell adhesion and eosinophil recruitment.^7^, ^129^ The mechanisms underlying these context‐dependent immunological effects of CGRP remain to be fully elucidated. Further investigations are required to better delineate cell‐type‐specific CGRP‐RAMP1 signalling and its context‐dependent functional consequences in the TME, including the conditions that may shift its effects toward either pro‐inflammatory or pro‐tumorigenic outcomes, which may ultimately inform CGRP‐targeted therapeutic strategies.

##### SP

A series of studies have revealed the tumour‐promoting effect of SP, mainly by interacting with the neurokinin‐1 receptor (NK1R) on the cancer cells.

SP has been reported to promote cancer progression through direct effects on cancer cells. In colorectal, ovarian, and gastric cancer cells, SP‐NK1R signalling enhances tumour‐cell proliferation, migration and invasion, partly through MMP‐2, MMP‐9,^119^ or calcium signalling.^124^ SP also promotes angiogenesis by upregulating VEGF and VEGFR in ovarian cancer^119^, ^120^ models, whereas NK1R antagonists show anti‐angiogenic activity in pancreatic cancer^130^ models.

A recent breast cancer study in 2024^11^ elucidated a new mechanism by which SP facilitates cancer progression. SP was reported to induce death of TACR1‐high tumour cells, leading to ssRNA release and TLR7‐dependent oncogenic signalling in neighbouring cancer cells.

However, several observations complicate a simple pro‐tumour interpretation. SP pretreatment suppresses K1735 melanoma growth in an NK‐ and T‐cell‐dependent manner^125^ in a mouse model. In non‐cancerous inflammatory diseases, SP promotes pro‐inflammatory cytokine release, enhances T cell responses, inhibits Treg function, and supports T lymphocyte survival.^131^, ^132^, ^133^, ^134^, ^135^

In the 4T1 breast cancer model, RTX‐mediated denervation did not significantly reduce intratumoral SP, suggesting non‐neuronal sources of SP.^136^ In fact, SP has been detected in fibroblasts, keratocytes, immune cells, epithelial cells during inflammation.^137^ TAC1, which encodes SP, can also generate multiple peptide products, including neuropeptide A, K and Y,^138^ which may further diversify biological outcomes.

Some researchers suggested that the paradoxical role that SP plays during cancer progression may correlate with specific TME conditions, as SP/NK1R inhibition seemed to demonstrate an anti‐tumour effect in tumours with high immune infiltration while manifesting a pro‐metastasis property in low immune infiltration situations.^139^

Future research may elucidate the binary pro‐/anti‐tumour view of SP/NK1R by establishing a spatiotemporal, context‐dependent framework, deciphering how immune‐infiltration thresholds, cellular sources of SP, and alternative TAC1‐derived peptides orchestrate its switch from an endogenous immune adjuvant to a metastatic driver.

#### 3.2.2 Emerging non‐canonical mediators

High mobility group box 1 (HMGB1) is traditionally recognized as a damage‐associated molecular pattern (DAMP) released following tissue injury.^140^ However, emerging evidence extends its function beyond passive danger signalling, suggesting that HMGB1 can also be actively released by sensory neurons and participate in neuroimmune regulatory circuits. Optogenetic activation of nociceptors triggers direct HMGB1 release,^141^ indicating that sensory neurons can secrete HMGB1 following activation. In cancer, HMGB1 has been implicated in tumour‐promoting effects through TLR4‐mediated expansion of myeloid‐derived suppressor cell (MDSC)^142^ and receptor for advanced glycation end products (RAGE)‐mediated enhancement of tumour invasion and inflammatory signalling, and is linked to adverse prognosis across multiple malignancies.^143^ However, emerging evidence indicates context‐dependent anti‐tumour effects of HMGB1. Although HMGB1 has been associated with anti‐apoptotic effects and chemotherapy resistance,^144^ it may also enhance chemotherapy effect by facilitating an apoptosis‐to‐senescence transition in cancer cells,^145^ and may improve immunotherapy responses by promoting CD8^+^ T cell recruitment.^146^

IL‐6 has emerged as a neuron‐associated immunomodulatory mediator. Melanoma and head and neck carcinoma mouse models have demonstrated that tumour‐derived small EVs can activate DRG neurons, inducing IL‐6 and SP release, contributing to CD8^+^ T‐cell dysfunction and MDSC accumulation.^147^

Glutamatergic signalling represents another emerging axis of sensory neuron–tumour communication. Nociceptor activation drives glutamate release under physiological conditions, although it should be emphasized that this evidence derives exclusively from CNS studies.^148^, ^149^ In PDAC, sensory fibres form pseudo‐synaptic contacts with cancer cells enriched in the GRIN2D/NMDAR2D (subunits of NMDA receptor), creating localized signalling domains where neuron‐derived glutamate may promote tumour proliferation and dissemination.^150^ Together, these observations suggest a model in which glutamate functions as a neurotransmitter‐like signal in neuron‐tumour interfaces, although whether sensory neuron activation directly modulates this axis in vivo under cancer conditions via neuron‐derived glutamate remains to be fully established.

Somatostatin represents an inhibitory neuropeptidergic output of sensory neurons. Peripheral nociceptor activation induces somatostatin release, which exerts anti‐inflammatory and antinociceptive effects in peripheral tissues,^151^ though the direct evidence has come from non‐cancerous disease (arthritis). In cancer biology, somatostatin signalling is most clearly characterized in somatostatin receptor (SSTR)‐positive neuroendocrine tumours, where somatostatin analogues have demonstrated anti‐tumour effects.^152^ These data suggest that somatostatin‐SSTR signalling may represent a therapeutically actionable pathway in tumour biology. However, current evidence does not support a model in which sensory neuron‐derived somatostatin influences tumour progression, which may need further exploration.

Importantly, although individual molecular pathways of these emerging targets are supported by experimental evidence, a fully integrated in vivo model linking sensory neuron activation, specific mediator release patterns, and coordinated effects on tumour progression remains incompletely defined.

### 3.3 Systemic effect of cancer‐related pain

#### 3.3.1 Chronic pain, HPA/SAM axis and cancer progression

Stress and pain are positively correlated. Patients with chronic pain often report worsened symptoms under stress, whereas stress reduction strategies can alleviate pain.^153^ Trans‐neuronal tracing showed that sensory neurons innervating the TME may activate brain circuits associated with pain and stress processing in orthotopic OSCC mouse models.^154^

The relationship between stress and cancer has been widely recognized.^155^, ^156^, ^157^ The hypothalamic‐pituitary‐adrenal (HPA) and sympathetic‐adrenomedullary (SAM) axes, together with glucocorticoids and catecholamines, are key pathways through which stress influences oncogenic processes, including cancer cell survival, immune evasion and angiogenesis, thereby promoting disease progression.

#### 3.3.2 Pain, central nuclei and cancer progression

Beyond stress, pain may influence cancer progression through CNS nuclei that modulate peripheral immunity, reshape the TME via sensory‐sympathetic/vagal circuits, or directly affect cancer cell behaviour. Brain–body neural circuitry is increasingly recognized in tumour pathogenesis and cancer progression.^106^

##### Peripheral immune responses

A recent study^158^ demonstrated that peripheral neuropathic pain may modulate the acetylcholine‐releasing neurons in the dorsal motor nucleus of the vagus (DMV) in mouse models, which directly innervate the spleen. During acute pain, nociceptive signals were transmitted to primary somatosensory cortex (S1) via the spinal cord and thalamus, and glutamatergic neurons in S1 predominantly activated DMV neurons to enhance splenic Th2 responses. Under chronic pain, a subset of GABAergic neurons in the central amygdala (CeA) were activated and predominantly inhibited DMV neurons, thereby suppressing Th2 responses. Beyond Th2‐mediated immunity, the DMV and vagus nerves may also regulate inflammation^159^ and anti‐tumour immunity.^160^

Hypothalamic paraventricular nucleus (PVN) may also be involved in pain–immune regulation. Tyrosine hydroxylase‐expressing PVN neurons modulated peripheral leucocyte mobilization during inflammatory pain by regulating corticosterone release.^161^ Furthermore, inhibitory GABAergic neurons within the PVN of the hypothalamus may contribute to CRP, as their ablation aggravated visceral pain in a pancreatic cancer mouse model, while chemo‐genetic activation of these neurons alleviated pain.^162^ As a key autonomic centre,^163^ the PVN contains stress‐related neuronal populations that may also influence cancer progression.^164^

In addition, sensory nerve injury has been associated with changes in the anterior cingulate cortex (ACC)‐ventral tegmental area feedback loop, leading to anxiety‐ and depression‐like behaviours in a mouse model.^165^ Glutamatergic neurons in the S1 and GABAergic neurons in the CeA were likewise affected and involved in modulating splenic immune responses.^166^

##### Sensory–sympathetic and sensory–vagal circuits

Sensory neurons can detect signals within the TME and, through central circuits involving brainstem nuclei, reflexively modulate sympathetic or vagal outflow to reshape the TME. Several neuron–immune–cancer circuits involving sensory neurons have been identified in several studies and reviews.^167^, ^168^, ^169^, ^170^, ^171^ Specifically, sensory nerves detect inflammatory mediators, MMPs and DAMPs within the TME, initiating nociceptive signalling that ascends via multiple nuclei and pathways to the insular cortex (InsCtx).

In sensory–sympathetic circuits, ascending signals project to the CeA and PVN and subsequently to the lateral paragigantocellular nucleus (LPGi), driving sympathetic outflow and modulating the microenvironment through norepinephrine (NE). In sensory–vagal circuits, signals are transmitted to the DMV / rostral ventrolateral medulla (RVLM), and then to vagus nerves, which modulate the microenvironment via acetylcholine (ACh). Notably, the correlations between sympathetic/parasympathetic nervous systems and cancer progression are well recognized.^172^

##### Biological behaviour of the cancer cells

In a head and neck cancer mouse model, the CeA and parabrachial nucleus (PBN) showed increased activity (indicated by cFos or △FosB) in tumour‐bearing mice versus controls, which could be partially suppressed by genetic TRPV+ sensory fibre ablation or analgesics,^154^ implying a tumour–brain circuit activated by tumour‐related nociceptive signalling. Interestingly, genetic TRPV+ sensory neuron ablation also inhibited cancer proliferation. Beyond its role in emotion and fear,^173^ the central median amygdala (CeM) may also be involved in pain processing, as trigeminal and spinal nociceptive signals ultimately converge there.^138^ Pharmacogenetic and chemogenetic CeM inhibition significantly alleviated anxiety‐like behaviours and reduced tumour growth in a breast cancer mouse model,^174^ implying a direct connection between brain nuclei and tumours that may promote cancer progression.

Collectively, these findings delineate a network in which peripheral pain signals modulate CNS nuclei, which in turn influence peripheral immunity or cancer cell behaviour, coupling nociceptive input to systemic responses that may affect cancer progression. Although some studies used conventional pain models, the role of CNS nuclei in linking pain and cancer progression remains biologically plausible given the close interplay between immunity and cancer progression.

**FIGURE 2:**
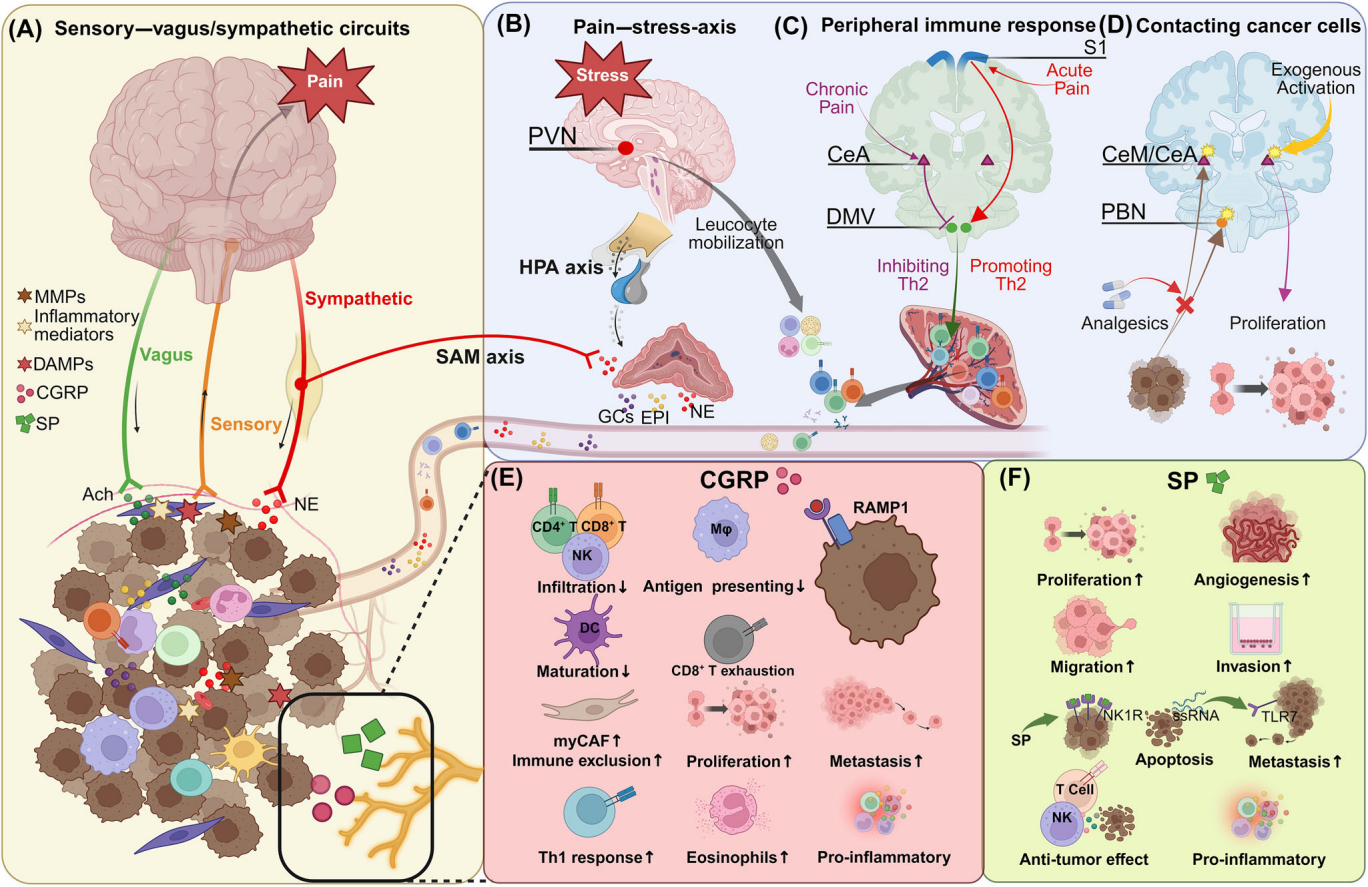
CRP may influence cancer progression. (a) Sensory nerve endings detect stimuli within the TME, ultimately modulating sympathetic and vagal outflow through series of central nuclei. (b) Pain is tightly coupled with stress. Persistent pain may activate the HPA axis and the SAM axis, leading to elevated systemic levels of glucocorticoids and catecholamines. (c) Acute nociceptive signals are transmitted to the S1. Glutamatergic neurons in S1 may subsequently activate cholinergic neurons in the DMV, driving vagal‐mediated splenic signalling that favours a Th2 response in the spleen. In contrast, chronic pain engages the activation of GABAergic neurons within the CeA, which appears to exert inhibitory control over DMV cholinergic activity, thereby dampening the splenic Th2 response. (d) Peripheral tumour (head and neck cancer) induces neuronal activation in CeM and PBN, which could be attenuated by analgesic interventions. Exogenous stimulation of CeM has been shown to accelerate peripheral tumour growth in preclinical models (breast cancer). (e) CGRP: undermines anti‐cancer immunity; promotes cancer proliferation and metastasis; pro‐inflammatory role in non‐cancerous settings. (f) SP: facilitates cancer proliferation, migration and invasion; promotes angiogenesis; promotes metastasis by SP‐apoptosis‐ssRNA‐TLR7 axis; anti‐tumour effect and proinflammatory role. CeA, central amygdala; CeM, central median amygdala, belongs to CeA; CGRP, calcitonin gene‐related peptide; DMV, dorsal motor nucleus of the vagus; EPI, epinephrine; HPA axis, hypothalamic‐pituitary‐adrenal axis; NE, norepinephrine; NK1R, neurokinin‐1 receptor; PBN, parabrachial nucleus; PVN, paraventricular nucleus; RAMP1, receptor activity‐modifying protein 1; SAM axis, sympathetic–adrenal medullary axis; SP, substance P; S1, primary somatosensory cortex; TME, tumour microenvironment. Original figure created with BioRender.com.

Figure 2 illustrates potential mechanisms by which CRP modulates tumour progression.

## 4 THERAPEUTIC DILEMMAS AND TRANSLATIONAL OPPORTUNITIES AT THE NOCICEPTION‐ONCOLOGY INTERFACE

### 4.1 Crosstalk between anti‐cancer and analgesic therapy

#### 4.1.1 Opioids and cancer progression: A dual‐edged sword

The majority of cancer patients suffer from pain, and about 80% of those with advanced disease need opioid therapy for adequate analgesia.^175^, ^176^ However, a growing body of research has demonstrated that beyond its analgesic effects, opioids exert complex effects on cancer progression. The specific role of opioids, whether tumour‐promoting or tumour‐inhibitory, remains controversial.^177^, ^178^, ^179^, ^180^

Opioids exert their analgesic effects by binding to and activating the opioid receptors, mainly the μ opioid receptor (MOR).^175^, ^181^ In addition to the nervous system, the MOR is also expressed in various cancer types including oesophageal cancer,^182^ gastric cancer,^183^ lung cancer,^14^ laryngeal cancer.^184^ Besides, compared with normal tissues, the cancer tissues seem to exhibit higher MOR expression,^185^, ^186^ which positively correlates with poor prognosis in multiple cancer types.^183^, ^185^, ^187^

In a retrospective study focusing on patients with NSCLC, continued post‐lobectomy opioid administration was considered an independent predictor of poorer overall and cancer‐specific survival.^14^ Similar results were identified in series of studies concerning different cancer types, such as pancreatic cancer,^188^ breast cancer,^13^ oesophageal squamous cell carcinoma (ESCC),^189^ where opioid use was associated with increased recurrence or poorer OS. However, retrospective studies in breast cancer^190^ and NSCLC^191^ with similar designs reported no significant difference between opioid dose and patients’ survival. Interestingly, a retrospective study also suggested that tramadol may help reduce the recurrence and mortality in breast cancer.^192^

Mechanistically, opioids may contribute to cancer progression via various mechanisms, including angiogenesis,^193^, ^194^, ^195^, ^196^, ^197^, ^198^ immune regulation,^199^, ^200^, ^201^, ^202^, ^203^, ^204^, ^205^, ^206^ direct promoting effect on cancer cells,^206^, ^207^, ^208^, ^209^ epithelial‐mesenchymal transition,^209^, ^210^, ^211^ and impaired efficacy of anti‐cancer therapies.^207^, ^212^, ^213^, ^214^, ^215^, ^216^ However, it should also be noted that opioids may also have a protective role against cancer progression potentially by non‐canonical effects on immune modulation,^217^, ^218^, ^219^, ^220^, ^221^ cancer biology^222^ and its secondary pharmacological effect on noradrenaline and serotonin reuptake,^223^, ^224^ particularly in non‐morphine opioids.

This controversy may be attributed to the widespread expression of opioid receptors on various cell types, including immune cells and tumour cells, as well as the existence of multiple receptor subtypes and various types of opioid receptor agonists, thereby adding complexity to the issue. Furthermore, while opioids affect cancer progression through diverse mechanisms, their primary function of pain relief may indirectly contribute to anticancer effects by alleviating pain‐inducing chronic stress responses,^153^ as stress is known to promote cancer progression.^155^, ^156^, ^157^

Table 4 summarizes bidirectional effects of opioids.

**TABLE 4:** Opioids and cancer progression.

Cancer types	Opioids/Receptor	Effect	Potential mechanism	Ref
TNBC	Morphine	Pro‐angiogenic	Increase c‐Myc and decrease TSP‐1 (inhibitor of angiogenesis)	^198^
Pancreatic cancer	Morphine	Pro‐tumour	Activating MOR on macrophage facilitate cancer cell proliferation in co‐culture	^206^
Ovarian cancer	Fentanyl	Promote migration and proliferation	Activate EGFR signalling	^207^
Bladder cancer	MOR agonist	Promote CTCs formation	MOR/AKT/Slug pathway	^208^
Pancreatic cancer	Morphine	Enhance proliferation and invasion	Increased stem markers, increased EPCAM and PDX‐1	^206^
Breast cancer	DOR	Promote metastasis	JAK1/2‐STAT3 signalling and increased Expression of EMT markers	^210^
Breast cancer	KOR	Facilitate migration	Upregulate EMT markers	^209^
CRC	MOR	Promote lymph node metastasis	PI3K/AKT signalling and EMT	^211^
NSCLC	M3G^a^	Facilitate growth and metastasis	1. PI3K signalling → elevates PD‐L1 on cancer cells 2. Impair CTL function	^213^
Ovarian cancer	Fentanyl	Resist chemotherapy	Activate EGFR signalling	^207^
Breast cancer	Morphine	Resist chemotherapy	Decreased ratio of Bax/Bcl‐2 mRNA	^216^
Lung cancer	Tramadol	Anti‐cancer	Preserve NK cell function	^217^, ^218^
Breast cancer	Tramadol	Anti‐cancer	Inhibit adrenergic signalling by noradrenaline and serotonin reuptake	^223^, ^224^
HCC	Butorphanol	Anti‐cancer	Suppress angiogenesis and migration by modulating MAPK pathway	^222^

Abbreviations: CTCs, circulating tumour cells; CRC, colorectal cancer; DOR, δ‐opioid receptor; EMT, epithelial‐mesenchymal transition; HCC, hepatocellular carcinoma; KOR, κ‐opioid receptor; MAPK, mitogen‐activated protein kinase; MENK, methionine enkephalin; MOR, μ‐opioid receptor; M3G, morphine‐3‐glucuronide; TNBC, triple‐negative breast cancer.

^a^ Long‐term use of morphine may result in abnormally elevated serum morphine‐3‐glucuronide (M3G) levels.

#### 4.1.2 Physical analgesic interventions and tumour progression

Apart from pharmacological interventions, some physical interventions for CRP therapy may also exert an impact on cancer progression.

Nerve ablation is a commonly employed clinical intervention for CRP, such as celiac plexus neurolysis in PDAC.^225^ However, the effect of sensory ablation on cancer progression remains under debate in different studies.^9^, ^101^, ^102^, ^103^, ^104^ In addition, a recent study published in 2024 suggested a skeptical attitude toward the efficacy of sympathetic nerve ablation in CRP management based on 17 studies involving 1025 cancer patients with abdominal or pelvic pain, but this approach may reduce the use of opioids.^226^ Therefore, whether sensory nerve ablation could represent a promising strategy for cancer therapy needs further exploration.

Electroacupuncture is another way to target CRP in traditional Chinese medicine, which has been proved by clinical trials and meta‐analysis.^227^, ^228^ Though the precise mechanism was not fully understood, it has been indicated in research that this intervention to relieve CRP could boost the anti‐cancer immunity in a mouse model with breast cancer,^160^ possibly through activating the vagus nerve and modulating inflammatory cytokines.

#### 4.1.3 Anti‐cancer therapies as contributors to cancer‐related pain

##### Chemotherapy

Chemotherapy‐induced peripheral neuropathy (CIPN) is characterized by degeneration of sensory nerve axons beginning distally and progressing retrogradely in cancer patients receiving chemotherapy, predominantly affecting the distal extremities, with mechanisms that are partly drug‐specific.^229^, ^230^

First, tubulin‐targeting agents may produce a distal sensory axonopathy. Mouse studies of anti‐tubulin chemotherapies have shown acute peripheral axonal injury with incomplete long‐term recovery.^231^ Second, mitochondrial and redox stress appear to be correlated with chemotherapy‐induced axonal degeneration.^232^ In breast cancer survivors with persistent paclitaxel‐induced neuropathy, mitochondrial dysfunction‐related pathways were differentially represented,^233^ while a zebrafish peripheral neuropathy model using Paclitaxel suggested ROS as upstream of MMP‐13‐dependent degeneration of unmyelinated axons.^234^ Third, ion‐channel hyperexcitability has been implicated in CIPN. For example, oxaliplatin slows NaV1.6 fast inactivation at hyperpolarized potentials, prolongs channel open times in DRG neurons, which may explain the cold‐induced hypersensitivity observed in human and mouse.^235^ Fourth, neuroimmune signalling may contribute to nociceptive sensitization. Paclitaxel induced IL‐6 synthesis in sensory neurons, while IL‐6‐deficient mice were protected from mechanical hypersensitivity and axonal pathology.

##### Radiotherapy

Radiotherapy plays a bidirectional role in CRP. On the one hand, it is recognized as a potential cause of treatment‐induced pain.^236^, ^237^ Pre‐clinical data indicated that radiation may upregulate TRPV1 channels in rodents, thereby lowering nociceptive thresholds and leading to oversensitivity of mechanical and heat allodynia.^238^ Radiation may induce direct axonal injury or elicit local inflammation via damage to neoplastic and surrounding normal tissue, culminating in perineural fibrosis and atrophy that further amplify pain intensity.^239^

On the other hand, radiotherapy may serve as an effective analgesic modality,^240^ achieving objective pain response rates of 60%–80% in clinical observations.^241^ The analgesic benefit was thought to reflect shrinkage of infiltrative tumour masses with decompression of pain‐generating structures.^242^ Radiotherapy has demonstrated effects in palliating pain arising from bone metastases^243^ and advanced HNSCC.^244^ Notably, radiotherapy in CRP also reduced the administration of opioids in HNSCC.^244^

##### Immunotherapy

Interestingly, even immunotherapy may have complex bidirectional influence on CRP. Nivolumab, an anti‐PD1 monoclonal antibody, was reported to trigger acute pain through microglial activation and upregulated glycolytic flux during bone tumour therapy with potential involvement of the microbiota.^245^ Another study investigating nivolumab similarly indicated a more sophisticated effect of immunotherapy on CRP, which observed that nivolumab induced mechanical and thermal pain at an early stage in mice, while exerting a long‐term effect of bone cancer pain relief by suppressing osteoclast genesis and thus preventing bone destruction.^246^ In addition, administration of anti‐PD‐1 therapy in the spinal trigeminal nucleus caudalis (Sp5C) of mice with oral cancer could significantly inhibit hyperalgesia, and reduced TNF‐α levels within the Sp5C were thought to be a potential mechanism.^247^

### 4.2 Emerging therapeutic strategies for cancer‐related pain

Given that cancer pain may influence cancer progression, which underscores its clinical significance, it is therefore of paramount importance to summarize current therapeutic advances in its management. Table 5 demonstrates the advances in CRP treatments.

**TABLE 5:** Advances in the management of CRP.

Module	Study type	Interventions / targets	Cancer types	Outcomes / effects	Ref
Psychological interventions	Clinical trail	PMR	Patients with head and neck cancers	The PMR group displayed significantly lower overall pain and muscle tightness than control group along with the timeline of multiple measurements (*p* < .01).	^248^
	Meta‐analysis	Mindfulness, hypnosis, yoga, guided imagery, and progressive muscle relaxation	Patients with cancer pain	Mind–body therapies may be effective in improving cancer pain, but the quality of the evidence is low.	^249^
	Meta‐analysis	MBIs	Patients with cancer pain	The efficacy of MBIs in reducing pain intensity among cancer patients was revealed in this meta‐analysis, albeit with a small effect size.	^250^
	Clinical trial	Cognitive restructuring	Patients with lung cancer, GI cancer, breast cancer and other cancers	Cognitive restructuring has significant impact in cancer patients on reducing pain and can ease problems related to pain	^251^
Nociceptive neuropeptides / alarmins	Preclinical study	CGRP receptor antagonist (Olcegepant)	Orthotopic oral cancer xenograft tumours in mice	CGRP antagonism reverses oral cancer nociception in preclinical oral cancer pain models.	^70^
	Preclinical study	CGRP receptor antagonism	Oral squamous cell carcinoma model	Targeting CGRP may serve as a potential therapeutic strategy in OSCC to reduce pain and improve therapeutic response	^252^
	Preclinical study	HMGB1 or RAGE (one receptor for HMGB1)	Breast cancer bone metastasis model	Neutralization of HMGB1 or antagonism against RAGE ameliorated breast cancer associated bone pain	^253^
	Preclinical study	NK1R antagonism using aprepitant	Lung cancer bone metastasis	The blockade of the SP‐neurokinin 1 receptor pathway effectively alleviated CIBP	^48^
NGF / sensory ablation	Clinical trial phase III	Anti‐NGF antibody tanezumab	Patients with bone metastasis	Tanezumab 20 mg met the primary efficacy endpoint at Week 8	^60^
	Preclinical study	Anti‐NGF and/or anti‐TNF	Lung cancer bone metastasis	Inhibition of NGF and/or TNF resulted in a significant prevented the development of secondary cutaneous heat hyperalgesia	^254^
	Clinical trial phase I	Intrathecal RTX	Refractory cancer pain localized to the abdomen and/or lower extremities	Intrathecal RTX is a single‐administration, opioid‐sparing analgesic in patients with intractable cancer pain	^255^
Angiogenesis	Preclinical study	VEGF‐A/VEGFR2	Lewis lung carcinoma bone metastasis	Mice after cancer cell injection demonstrated mechanical allodynia and thermal hyperalgesia, both of which were suppressed via anti‐VEGF‐A antibody or ZM323881	^67^
Inflammatory mediators	Systematic review of animal model	TNF‐α, IL‐1β, IL‐6, IL‐17, IL‐18, IL‐33	Bone cancer pain models	Targeting inflammatory cytokines consistently mitigated pain behaviours in multiple animal models	^37^
Central sensitization	Preclinical study	Spinal astrocytes and astrocyte‐derived IL‐17	Bone cancer pain model	Chemogenetic or pharmacological inhibition of spinal astrocytes effectively ameliorates bone cancer‐induced pain‐like behaviours	^93^
	Preclinical study	Microglia	Cancer‐induced bone pain	Intrathecal administration of 2‐DG effectively alleviated pain‐related behaviours and suppressed microglial activation and M1 polarization, restoring M2 phenotype.	^256^
	Preclinical study	Ferroptosis‐like cell death of spinal interneurons	Bone cancer pain model	Pharmacological inhibition of ferroptosis‐like cell death of spinal interneurons alleviates BCP in mice	^257^
	Preclinical study	GPR37 in DRG neurons	Bone cancer pain model	Activation of the GPR37 alleviates both acute and persistent pain in multiple mouse models of bone cancer	^258^

Abbreviations: BCP, bone cancer pain; CGRP, calcitonin gene‐related peptide; CIBP, cancer‐induced bone pain; DRG, dorsal root ganglia; GPR37, G protein‐coupled receptor 37; HMGB1, high mobility group box 1; MBIs, mindfulness‐based interventions; NGF, nerve growth factor; NK1R, neurokinin 1 receptor; OSCC, oral squamous cell carcinoma; PMR, progressive muscle relaxation; RAGE, receptor for advanced glycation end products; RTX, resiniferatoxin; SP, substance P; VEGF, vascular endothelial growth factor.

Psychological and behavioural interventions, including progressive muscle relaxation (PMR),^248^ cognitive reconstruction,^251^ and mindfulness‑based approaches,^250^ are increasingly recognized as adjuncts in cancer pain management, though definitive efficacy requires confirmation in larger‐scale studies.

Although pain‑related neuropeptides have garnered preclinical support as emerging therapeutic targets, clinical data in this area are still limited. CGRP signalling blockade, already validated for migraine,^259^, ^260^ has shown preclinical analgesic effects in oral cancer pain,^70^ while targeting the SP/NK1R axis demonstrated preclinical analgesic effects in CIBP models.^48^, ^261^

Targeting NGF‐related nociceptor sensitization and pathological sensory innervation represents a relatively advanced strategy. In a Phase III randomized trial of bone metastasis‐related cancer pain, tanezumab met the primary endpoint at Week 8, although long‐term efficacy and safety remain concerns.^60^ In parallel, intrathecal RTX targets TRPV1‐positive sensory neurons and has shown an opioid‐sparing analgesic signal after a single administration in patients with intractable cancer pain.^255^

Central sensitization may constitute another potential target for intervention. Modulating astrocyte or microglia activation has showed promising analgesic effects in CIBP models, possibly by regulating inflammation within the spinal cord.^93^, ^256^ Targeting a wide range of other targets, including VEGF‐A/VEGFR2^67^, inflammatory cytokines^37^ and HMGB1/RAGE signalling,^253^ have demonstrated pain‑relieving effects in animal models, but evidence from clinical studies are still lacking.

Cancer treatment‐induced pain requires etiologically specific management because chemotherapy‐, radiotherapy‐, and immunotherapy‐related pain arise from different neuropathic, tissue injury and immune‐inflammatory mechanisms.

For CIPN, duloxetine remains the major pharmacologic option with sufficient evidence,^262^ though its benefit is modest.^263^ A 2025 observational study^264^ suggested amitriptyline plus ketamine as a possible alternative. Non‑pharmacological interventions such as acupuncture and limb compression showed potential benefits in recent clinical trials,^265^, ^266^ but larger confirmatory trials are still needed.

Radiotherapy‐related pain should be divided into analgesic radiotherapy and pain caused by radiation toxicity. For painful bone or spinal metastases, palliative external‑beam radiotherapy remains an evidence‐based analgesic treatment for symptomatic bone metastases,^267^ while stereotactic body radiotherapy has shown higher complete response rates in a randomized phase 2/3 trial.^268^ For radiotherapy‑induced oral mucositis pain, doxepin mouthwash provides rapid pain relief^269^, ^270^ in a Phase 3 clinical trial.

Immunotherapy‐induced pain most often reflects immune‐related adverse events, such as inflammatory arthritis or myositis. Current guidance recommends severity‐based management, typically beginning with symptomatic treatment and escalating to corticosteroids or disease‐modifying therapy when needed, together with temporary interruption or discontinuation of immune checkpoint inhibitors in clinically significant cases.^271^, ^272^, ^273^ Observational data suggest that IL‐6 receptor inhibition may help control immune checkpoint inhibitor‐associated inflammatory arthritis,^274^ but direct evidence using pain intensity as primary endpoints remains lacking.

**FIGURE 3:**
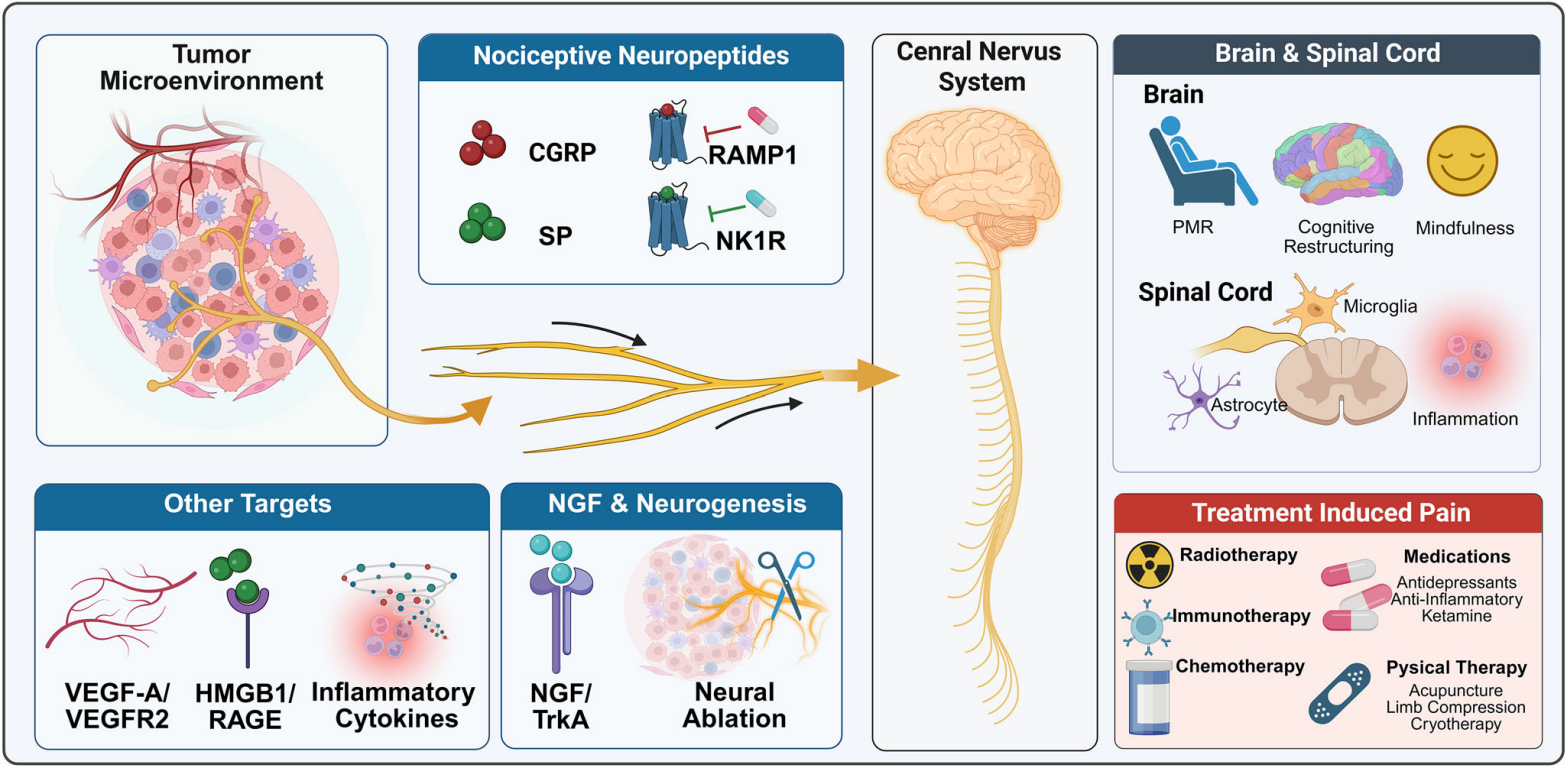
Potential targets or strategies for CRP management. Strategies showing preclinical or clinical effects include psychological interventions, glial cell modulation, neuropeptide (SP, CGRP) targeting, NGF/angiogenesis blockade, inflammatory cytokine inhibition, and management of treatment‑induced neuropathy. CGRP, calcitonin gene‐related peptide; HMGB1, high‐mobility group box 1; NGF, nerve growth factor; NK1R, neurokinin‐1 receptor; PMR, progressive muscle relaxation; RAGE, receptor for advanced glycation end products; RAMP1, receptor activity‐modifying protein 1; SP, substance P; TrkA, tropomyosin receptor kinase A; VEGF, vascular endothelial growth factor. Original figure created with BioRender.com.

Figure 3 demonstrates potential targets or strategies for CRP management.

### 4.3 Future directions for cancer therapy

**FIGURE 4:**
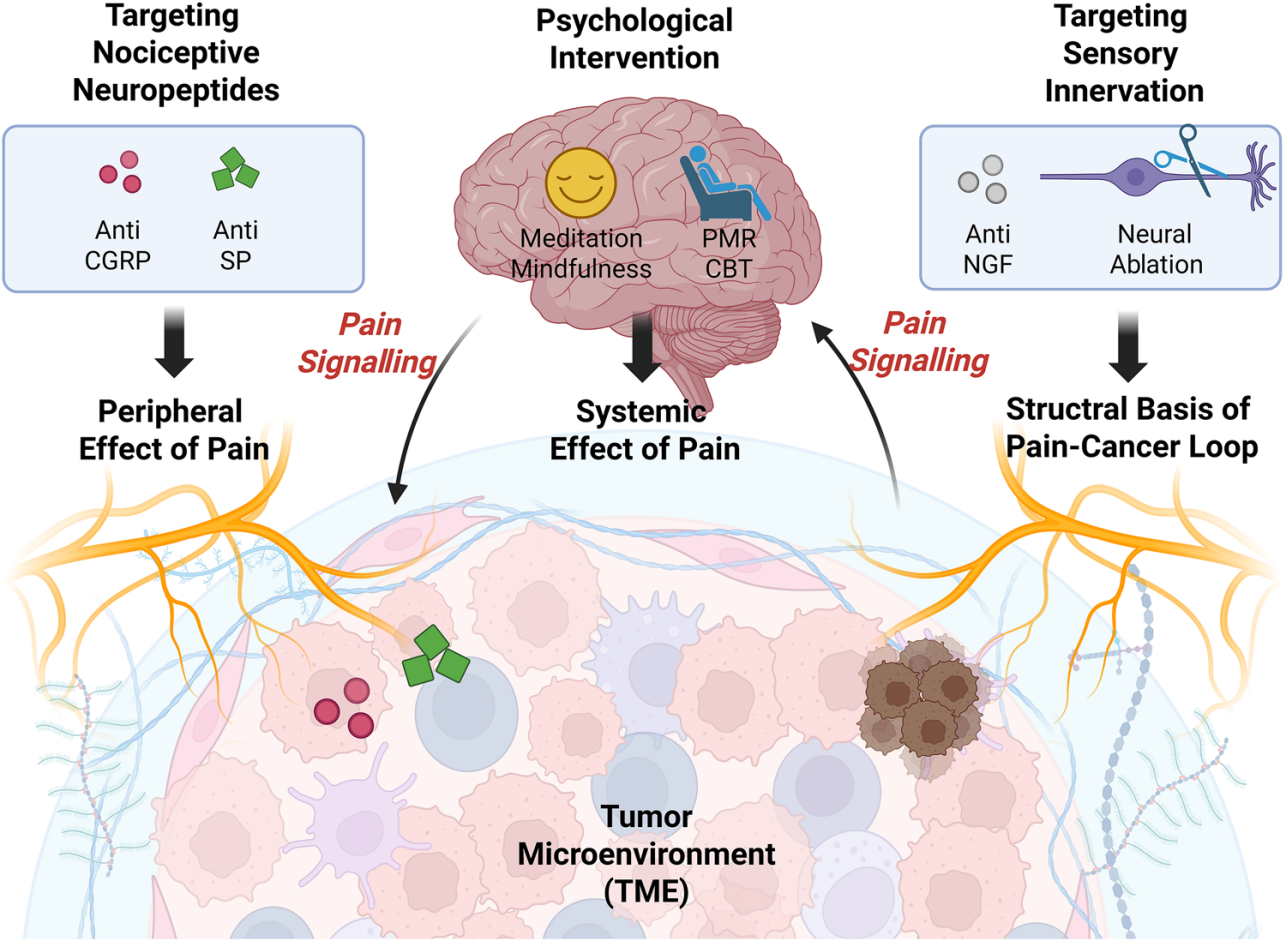
Potential future directions in cancer therapy based on pain–cancer crosstalk. Three future directions are proposed: (1) peripheral targeting of pain‑related neuropeptides, (2) central psychological interventions, and (3) structural targeting of NGF and sensory nerves. CBT, cognitive behavioural therapy; CGRP, calcitonin gene‐related peptide; NGF, nerve growth factor; PMR, progressive muscle relaxation; SP, substance P; TME, tumour microenvironment. Original figure created with BioRender.com.

Mechanistic studies on the interplay between CRP and cancer progression have highlighted a potential therapeutic paradigm in which targeting pain‐cancer crosstalk may influence cancer progression. Figure 4 illustrates possible future directions for cancer intervention by disrupting pain–cancer crosstalk.

#### 4.3.1 Psychological interventions

The association between pain and stress has been widely demonstrated in clinical observations^153^ and cancer‐bearing animal models.^154^ Several pain or stress‐responsive central nuclei may modulate nociception, peripheral immunity and potentially cancer progression.^174^ Key nodes include amygdala,^154^ PVN of the hypothalamus,^161^ glutamatergic neurons in S1^166^ and cholinergic neurons in DMV.^158^

Some psychological interventions targeting stress have been shown to alleviate pain or improve prognosis in cancer patients. A series of psychological interventions, including PMR,^248^, ^275^ guided imagery,^276^ mindfulness^277^ and meditation^278^ have been demonstrated to be effective in reducing pain, anxiety, depression and stress across patients with different cancer types. Among postoperative gastric cancer patients, an integrated intervention combining cognitive behavioural therapy (CBT), counselling, sleep education and training, as well as melatonin supplementation, significantly improved DFS and OS.^279^ Another randomized controlled trial (RCT) on breast cancer patients indicated a significantly lower risk of all‐cause mortality in the cognitive‐behavioural stress management (CBSM) group compared to control.^280^

#### 4.3.2 NGF and sensory innervation

An increased density of sensory nerve fibres in the peritumoral region has been identified across multiple cancer types.^11^, ^115^, ^116^ Such aberrant innervation is closely associated with enhanced nociceptive signalling and may also contribute to tumour progression.^116^, ^281^

NGF is one of the key factors involved in neurogenesis.^100^ In patients with pancreatic cancer, NGF signalling correlates with PNI, pain severity and prognosis.^61^, ^62^, ^63^, ^64^ In fact, anti‐NGF therapeutics have already reached a relatively advanced stage of development. Tanezumab, a monoclonal antibody targeting NGF, has been suggested in clinical settings, showing preliminary evidence and safety for cancer pain associated with bone metastasis at Week 8 compared to placebo.^60^

An early study in 2018 demonstrated that antagonism of NGF may reduce metastasis in a pancreatic cancer model.^282^ More recently, a study published in Cell (2026)^128^ provided compelling evidence that antagonizing NGF or targeting sensory innervation may exert direct anti‐tumour effects. In mouse model of breast cancer, tumour cells were shown to actively recruit sensory nerve fibres into the TME through NGF‐dependent mechanisms. This sensory innervation contributed to disease progression, at least in part, by engaging a CGRP‐myCAF‐mediated immune exclusion axis. Notably, both NGF antagonism and sensory ablation attenuated tumour progression, further indicating that disrupting NGF signalling and tumour‐associated sensory innervation may represent a viable therapeutic strategy. However, the definitive efficacy of targeting NGF signalling and sensory innervation requires confirmation in larger‐scale studies.

#### 4.3.3 Nociceptive neuropeptides

Clinical evidence suggests a significant association between CGRP signalling and patient prognosis. In OSCC, clinical studies have reported that tumour or circulating CGRP levels correlate with PNI, lymph node metastasis, pain severity and poorer survival outcomes.^283^, ^284^

Notably, pharmacological targeting of CGRP is already well established in other clinical settings. CGRP receptor antagonists have been widely adopted for the management of migraine, supporting the feasibility of therapeutic intervention of this target.^285^, ^286^

In line with this, preclinical studies have shown that CGRP receptor blockade exerts anti‐tumour effects across multiple malignancies, including melanoma,^101^ gastric cancer,^116^ breast cancer,^128^ pancreatic cancer,^56^ OSCC,^115^ HNSCC,^114^ etc., through directly inhibiting cancer cells or influencing immune cells and interstitial components within TME.

For example, treatment with the CGRP receptor antagonist rimegepant significantly reduced tumour burden and prolonged OS in a mouse model of pancreatic ductal adenocarcinoma by restoring NK cell function.^56^ In a murine melanoma model, blockade of CGRP receptor signalling suppressed tumour progression by reversing CD8^+^ T‐cell exhaustion.^101^

SP represents another neuropeptide with potential relevance to cancer progression. In colorectal adenocarcinoma, elevated NK1R expression, as assessed by immunohistochemistry, has been associated with advanced disease stage and poor differentiation.^287^

Clinically, NK1R antagonists such as aprepitant are already incorporated into antiemetic regimens for chemotherapy‐induced nausea and vomiting, as recommended by MASCC/ESMO guidelines, underscoring their safety and translational potential,^288^ but the guideline does not establish these drugs as anticancer therapies yet.

Consistent with their biological role, preclinical studies indicate that blockade of SP/NK1R signalling can suppress tumour progression in a variety of models, including breast cancer,^11^ prostate cancer,^289^ ESCC^290^ and even intracranial melanoma^291^ models. For example, targeting the SP receptor TACR1 with aprepitant significantly reduced tumour growth and metastasis in a breast cancer mouse model.^11^ In a murine model of ESCC, aprepitant significantly reduced tumour volume with reduced vascularized areas within tumour.^290^

Taken together, NGF, CGRP and SP signalling pathways display several key features of clinically relevant therapeutic targets, including clinical prognostic associations, pharmacological accessibility and supportive preclinical evidence. Importantly, accumulating loss‐of‐function evidence in both cellular and animal models further supports the specificity of these targets in cancer progression. For example, disruption of NGF/NGFR signalling has been shown to suppress tumour growth and metastasis in vivo^128^, ^292^, ^293^; depletion of CGRP signalling attenuates immune exhaustion and tumour growth in vivo^101^, ^112^; and knockdown or knockout of SP/NK1R signalling reduces tumour growth and metastasis.^11^ Representative loss‐of‐function studies for these targets are summarized in Table 6.

**TABLE 6:** CGRP, SP and NGF: Genetic loss‐of‐function evidence in cancer.

Targets	Gene knockout / knockdown design	Study design	Cancer type	Outcomes / effects	Ref
Ramp1 (component of CGRP receptor)	Ramp1^−^/^−^ CD8^+^ T	CD 8+ T cell (engineered) transplantation (engineered tumour‐bearing Rag1‐deficient mice)	Melanoma	Compared with wild‐type CD8+ T cells, Ramp1‐/‐ CD8+ T cells were protected from exhaustion	^101^
Calca (encoding CGRP)	global Calca knockout mouse	Tumour transplantation (engineered mice)	OSCC	In tumour‐bearing CGRPKO mice, there is a significant reduction in tumour size over time compared to wildtype mice	^112^
Ramp1 (component of CGRP receptor)	Ramp1‐knockout gastric cancer cells	Tumour transplantation (engineered cancer cells)	Gastric cancer	Knockout of Ramp1 in ACKP cells significantly inhibited tumour growth	^116^
Tacr1 (receptor of SP, aka. NK1R)	Lentiviral shRNA‐mediated silencing to deplete Tacr1 in 4T1 cancer cells	Cell experiment	Breast cancer	TACR1 depletion significantly reduced 4T1 spheroid invasion and proliferation in vitro and 4T1 tumour growth and metastasis in vivo	^11^
Tac1 (encoding precursor neuropeptide for SP and neurokinin A)	Tac1‐null mice	Tumour transplantation (engineered cancer cells)	Breast cancer	Mice lacking host‐derived SP (Tac1‐null) exhibited significantly reduced orthotopic tumour growth and metastasis across multiple syngenetic breast cancer models	^11^
Tacr1 (receptor of SP, aka. NK1R)	transfecting NCI‐H1944 and A549 cells with specific shRNA targeting NK1R	Cell experiment	NSCLC	The proliferation and migrative cell number were attenuated compared with un‐transfected and control‐shRNA groups	^294^
NGFR	NGFR knockout in T cells	T cell (engineered) and cancer cell coculture	Melanoma	Knockdown of NGFR promoted caspase‐dependent apoptosis upon T cell encounter	^295^
NGF	NGF knockout in MHCC97H cells (liver cancer cells with resistance to lenvatinib)	Tumour transplantation (engineered cancer cells)	Liver cancer	NGF knockout significantly suppressed tumour growth under lenvatinib treatment, while tumour growth rates in untreated mice were comparable between NGF‐KO and control groups	^293^
NGFR	Transfecting MDA‐MB‐231 cells with shRNA targeting NGFR	Tumour transplantation (engineered cancer cells)	Breast cancer	ShNGFR significantly reduced tumour burden of lung colonies	^292^
NGF	Transfecting 4T1 cells with shRNA targeting NGF	Tumour transplantation (engineered cancer cells)	Breast cancer	Significant inhibition of tumour growth in the shNgf groups was observed	^128^

Abbreviations: CGRP, calcitonin gene‐related peptide; NGF, nerve growth factor; NK1R, neurokinin 1 receptor; NSCLC, non‐small cell lung carcinoma; SP, substance P.

**FIGURE 5:**
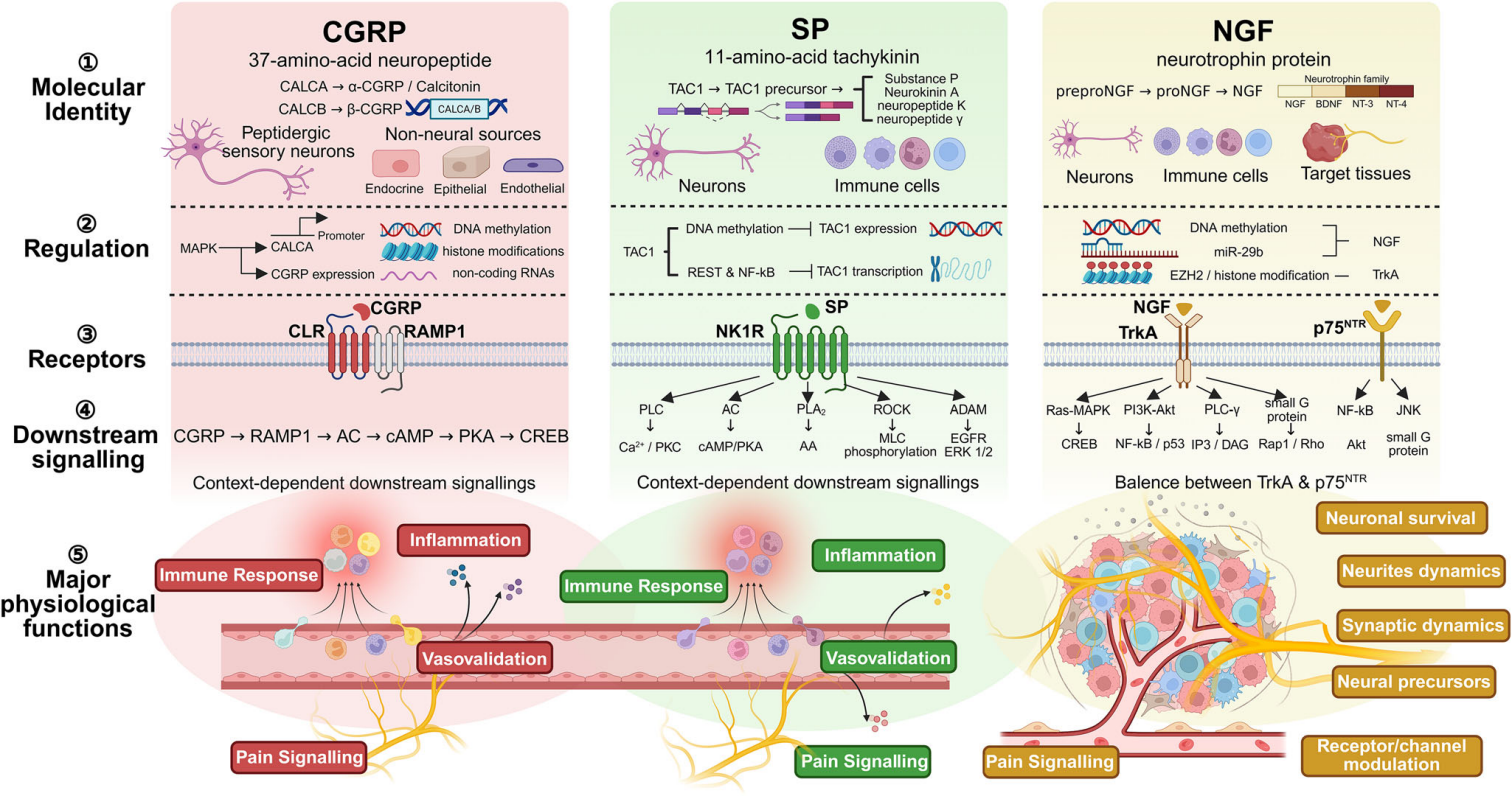
Multidimensional characterization of target molecules. CGRP, SP and NGF are three target molecules with therapeutic and translational potential in anti‐cancer treatment. Under physiological conditions, CGRP and SP mainly function in regulating inflammatory immunity, pain signalling and vasodilation, whereas NGF primarily plays roles in neural development, survival, neural signal transmission, neurite remodelling, and pain signalling. This figure presents information on these targets across five dimensions: molecular characteristics, regulation, receptors, downstream signalling and major physiological functions. AA, arachidonic acid; AC, adenylyl cyclase; ADAM, a disintegrin and metalloproteinase; BDNF, brain‐derived neurotrophic factor; cAMP, cyclic adenosine monophosphate; CLR, calcitonin receptor‐like receptor; CGRP, calcitonin gene‐related peptide; CREB, cAMP response element‐binding protein; DAG, diacylglycerol; EGFR, epidermal growth factor receptor; ERK, extracellular signal‐regulated kinase; EZH2, enhancer of zeste homolog 2; IP3, inositol trisphosphate; JNK, c‐Jun N‐terminal kinase; MAPK, mitogen‐activated protein kinase; MLC, myosin light chain; NGF, nerve growth factor; NK1R, neurokinin‐1 receptor; NT, neurotrophins; PLA2, phospholipase A2; PLC, phospholipase C; PKC, protein kinase C; RAMP1, receptor activity‐modifying protein 1; REST, RE1‐silencing transcription factor; ROCK, Rho‐associated coiled‐coil containing protein kinase; SP, substance P. Original figure created with BioRender.com.

Although early‐phase clinical studies exploring the anti‐cancer potential of these pathways are emerging, the current evidence remains preliminary. Definitive conclusions regarding their therapeutic efficacy will require further validation in larger, well‐designed clinical trials. Figure 5 presents relatively comprehensive information regarding the genetic encoding, epigenetic regulation and biological functions of these targets.

To sum up, preclinical studies have demonstrated the therapeutic potential of multiple targets based on mechanisms within CRP and cancer progression. Preliminary clinical studies are also gradually being initiated, although definitive results have not yet been obtained and many investigations remain in their early stages.

Relevant clinical trials are summarized in Table 7.

**TABLE 7:** Clinical trials targeting pain and progression in cancer.

Target	Interventions	Cancer Types	Outcomes / Measures	Status	Ref
NGF	Tanezumab / placebo	Bone metastases	Tanezumab effectively alleviated pain in bone metastases	Finished	NCT02609828
	Tanezumab / placebo	Schwannomatosis	Pain levels, advert events, orthostatic hypotension	Ongoing	NCT04163419
	Tanezumab / placebo + opioids	Bone metastases	Tanezumab showed better effect in patient with higher baseline pain or low opioids use, though no significant efficacy was identified	Finished	NCT00545129 NCT00830180
CGRP	Rimegepant^a^ + anti‐PD‐1	Colorectal cancer liver metastasis	ORR, PFS, OS, DCR, advert events	Ongoing	NCT06999252
	Erenumab^a^ / IL‐6 antagonist	Schwannomatosis	Pain intensity, advert events, orthostatic hypotension	Ongoing	NCT05684692
SP	Temozolomide combined with 9 repurposed drugs, including aprepitant^b^	Recurrent glioblastoma	Patients showed reduction of contrast enhancement on MRI Dose, PFS, OS (not published yet)	Finished	NCT02770378

Abbreviations: CGRP, calcitonin gene‐related peptide; DCR, disease control rate; NGF, nerve growth factor; PFS, progression‐free survival; ORR, objective response rate; OS, overall survival; SP, substance P.

^a^ Rimegepant, Erenumab: CGRP receptor antagonist

^b^ Aprepitant: NK1R (the receptor for SP) antagonist

## 5 CONCLUSIONS AND FUTURE PROSPECTIVE

In summary, CRP and tumour progression are not linked in a simple unidirectional way; rather, they engage in a dynamic, bidirectional circuit mediated by multiple mechanisms in neuroimmunology and cancer neuroscience. On the one hand, cancer progression promotes CRP through tissue destruction, inflammation, nerve injury and TME remodelling. On the other hand, CRP is not merely a passive symptom. Nociceptive neuropeptides (e.g., CGRP, SP), activation of pain–stress axis, central nuclei modulation, sensory–sympathetic/vagal circuits and subsequent alterations in peripheral immunity may influence tumour biology. Importantly, this bidirectional interplay extends to a treatment perspective, where analgesic interventions may inadvertently affect tumour progression, while anticancer therapies can influence pain. Several questions remain unresolved, such as the controversial role of sensory neurons during cancer progression and whether opioids carry pro‐tumorigenic risks. In the future, more clinical studies with larger sample size based on interplays between CRP and cancer progression with pre‐clinical evidence is anticipated to be conducted. Deeper investigations should be made to further elucidate the mechanistic interrelationships between them to identify novel therapeutic opportunities tackle CRP and cancer progression.

## AUTHOR CONTRIBUTIONS

Junzhe He, Yu Zhang, Peiwei Chai and Renbing Jia wrote and revised abstract. Junzhe He, Yu Zhang and Qian Liu wrote and revised ‘Cancer‐Related Pain: Peripheral Nociception and Central Sensitization’. Junzhe He, Qian Liu and Biao Yan wrote and revised ‘Pain as a Modulator of Malignancy: Deciphering the Afferent Neuro‐Tumour Signalling Axis’. Junzhe He, Yu Zhang and Biao Yan wrote and revised ‘Therapeutic Dilemmas and Translational Opportunities at the Nociception‐Oncology Interface’. Peiwei Chai critically revised the manuscript. Junzhe He, Yu Zhang, Peiwei Chai and Renbing Jia designed the structure of the review article, supervised the analysis and drafted the manuscript. All authors discussed and approved the final manuscript.

## CONFLICT OF INTEREST STATEMENT

The authors declare no conflicts of interest.

## ETHICS APPROVAL

The authors have nothing to report.

## Data Availability

The authors have nothing to report.
